# Using deep learning and molecular dynamics simulations to unravel the regulation mechanism of peptides as noncompetitive inhibitor of xanthine oxidase

**DOI:** 10.1038/s41598-023-50686-0

**Published:** 2024-01-02

**Authors:** Yi He, Kaifeng Liu, Fuyan Cao, Renxiu Song, Jianxuan Liu, Yinghua Zhang, Wannan Li, Weiwei Han

**Affiliations:** 1 Key Laboratory for Molecular Enzymology and Engineering of Ministry of Education, School of Life Sciences, Jilin University, Qianjin Road 2699, Changchun, 130012 China; 2 Jilin Academy of Chinese Medicine Sciences, Chuangju Road 155, Changchun, 130012 China; 3 Edmond H. Fischer Signal Transduction Laboratory, School of Life Sciences, Jilin University, Qianjin Road 2699, Changchun, 130012 China

**Keywords:** Computational biophysics, Molecular biophysics, Diseases, Endocrine system and metabolic diseases

## Abstract

Xanthine oxidase (XO) is a crucial enzyme in the development of hyperuricemia and gout. This study focuses on LWM and ALPM, two food-derived inhibitors of XO. We used molecular docking to obtain three systems and then conducted 200 ns molecular dynamics simulations for the Apo, LWM, and ALPM systems. The results reveal a stronger binding affinity of the LWM peptide to XO, potentially due to increased hydrogen bond formation. Notable changes were observed in the XO tunnel upon inhibitor binding, particularly with LWM, which showed a thinner, longer, and more twisted configuration compared to ALPM. The study highlights the importance of residue F914 in the allosteric pathway. Methodologically, we utilized the perturbed response scan (PRS) based on Python, enhancing tools for MD analysis. These findings deepen our understanding of food-derived anti-XO inhibitors and could inform the development of food-based therapeutics for reducing uric acid levels with minimal side effects.

## Introduction

Gout, a significant health threat, arises from excessive intake of purine-rich foods and alcohol, leading to uric acid accumulation in body tissues^1,2^. Hyperuricemia, gout's precursor, is linked to cardiovascular, kidney, and metabolic disorders^3^. Xanthine oxidase (XO) plays a crucial role in this process, also contributing to oxidative stress-related diseases^4–6^. Current XO-targeting drugs like allopurinol and febuxostat have safety concerns^7^. Therefore, finding safe, effective natural XO inhibitors is vital^8^. Recent research has identified XO inhibitory peptides from foods like walnut, rice, tuna and bonito have been reported^9–12^. Specifically, two new peptides, ALPM and LWM, derived from whey protein isolate (WPI) hydrolysates, have shown promise, with IC50 values of 7.23 ± 0.22 and 5.01 ± 0.31 mM, respectively. Both exhibit non-competitive inhibition modes^13^. Allosteric inhibition is often more specific than orthosteric or competitive inhibition at the active site, theoretically reducing off-target interactions that are commonly associated with side effects. Peptides especially Whey-derived-peptides can be added into dairy product such as yogurt and milk powder, so that we can develop food with anti-gout property.

It is an interesting and important proposition to explore how binding events of non-competitive inhibitors affect the interactions of protein active pockets with substrates^14–16^. Computational approaches for the analysis of allosteric coupling provide affordable opportunities to predict mutations or small molecule combinations to affect the active center^17,18^. Gaussian Network Models (GNM)^19^ and Anisotropic Network Models (ANM)^20^ are widely utilized to represent and analyze Elastic Network Models (ENM)^21^. For predicting key residues in allosteric transitions, Perturbation Response Scanning (PRS)^22–24^ applies isotropic perturbations to each residue, employing Linear Response Theory (LRT)^25^ to derive the fluctuation response profile of the entire network. Additionally, Constraint Network Analysis (CNA)^26^ is employed on network ensembles from MD trajectories to calculate neighbor stability maps and evaluate long-range effects of dynamic allostery using rigidity theory. The advent of deep learning, particularly the unsupervised Neural Relational Inference (NRI)^27^ model, offers novel approaches in studying allosteric regulations. The NRI-MD^28^ model, an adaptation of the NRI algorithm, excels in identifying distal protein residue interactions.

Molecular dynamics simulation is a strong method to investigate conformational changes of proteins, protein folding, protein–ligand binding, etc.^29,30^ In this study, we conducted 200 ns classical molecular dynamics simulation after global molecular docking, we also utilized deep learning method NRI-MD^28^ to reveal allosteric pathways, and explored the specific mechanism of non-competitive inhibition of XO by two different peptide inhibitors, including differences in peptide structure and different effects on protein conformations. This study may provide clues for the rational design of peptide inhibitors for XO.

## Materials and methods

### System preparation

**Figure 1:**
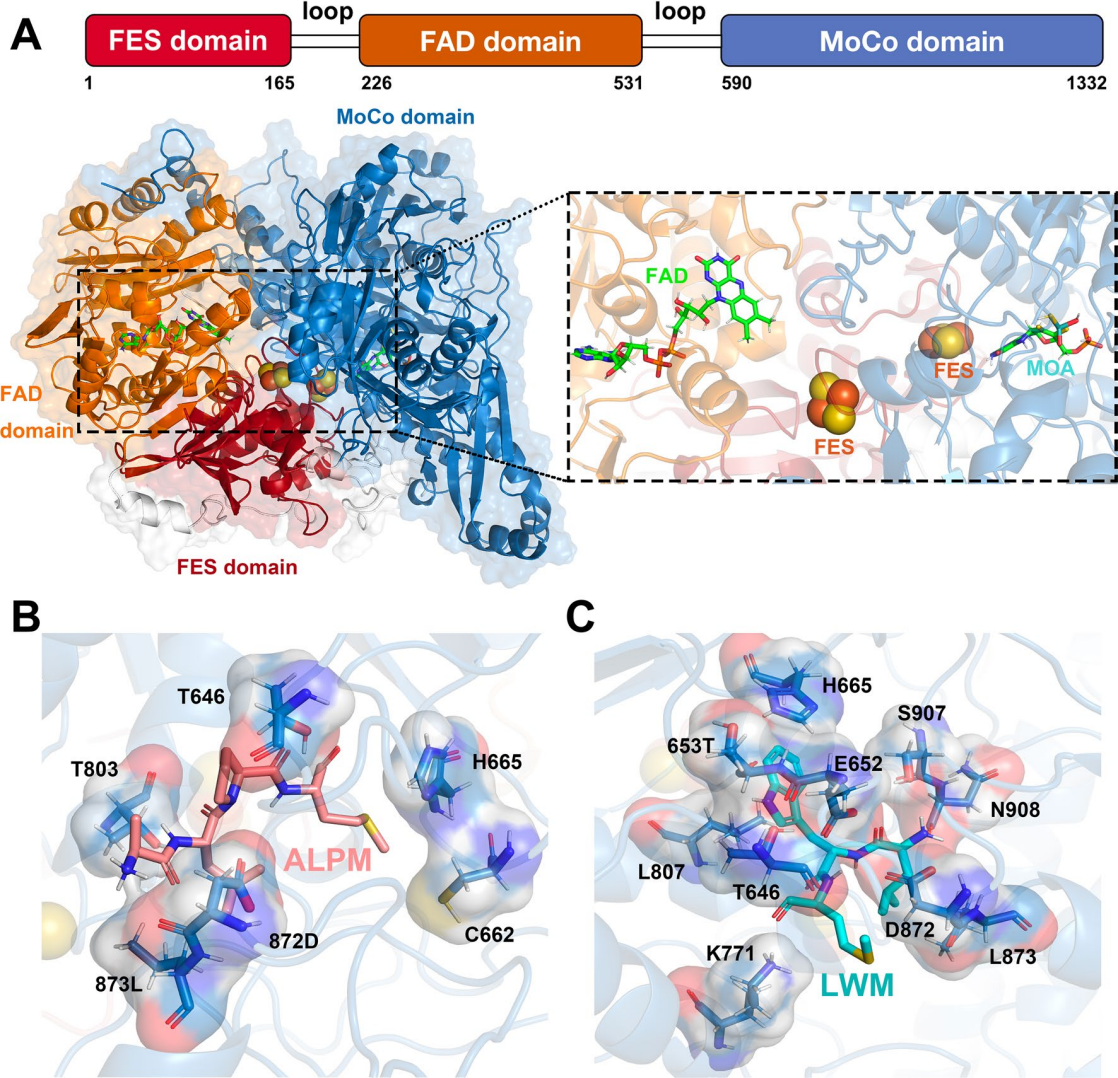
(**A**) 3D structure of one subunit of XO, the close-up represents the XO’s active region. (**B**) Binding pocket of ALPM. (**C**) Binding pocket of LWM.

Comparative modeling of XO's 3D structure and incorporation of the missing residues were performed on the basis of bovine milk xanthine oxidoreductase (PDB ID: 3BDJ)^31^ (1442 residues) using MODELER 10.1^32,33^, the domains and the structure were shown in Fig. 1A. The two peptides (ALPM and LWM) were constructed using Pymol. Next, we used Gaussian 16^34^ to optimize the structures of the two dipeptides at the level of B3LYP/6-31G* in order to obtain the optimal conformations for subsequent molecular docking. We utilized Autodock Vina 4.2^35^ for global molecular docking^36–38^, since both inhibitors are non-competitive inhibitors^39^, docking poses to active sites were ignored, and the lowest energy structure was selected as the initial structures for MD simulations, which can be seen in Fig. 1B and C. The Amber FF14SB force field^40,41^ were used for proteins, and the TIP3P water model^42,43^ was added for these systems. The parameterization protocol applied for the flavin adenine dinucleotide (FAD), molybdenum cofactor (MOA) and Fe_2_S_2_ cluster (FES) have been described previously^44,45^.

To avoid edge effects, periodic boundary conditions were given to the system during the simulation duration. The distance between the solute surface and the box was set to 12 Å. Since the charge in the initial reaction system is not zero, it was necessary to add Cl^-^ to the system in the initial stage of reaction simulation.

Systems under study were designated as follows: ALPM (Ala–Leu–Pro–Met) for XO–ALPM, LWM (Leu–Trp–Met) for XO–LWM, and Apo for XO without inhibitors.

### Molecular dynamics simulations

In our experiment, PMEMD.CUDA module^46^ of Amber 22 software^47^ was used to simulate the systems with 200 ns molecular dynamics simulations. All the bonds involving hydrogen atoms were constrained using the SHAKE algorithm^48^. The particle mesh Ewald (PME) algorithm^49^ was used to handle non-bonded electrostatic interactions, and the cut off was set to 8 Å.

After the systems were constructed, energy minimization for the three systems was carried out to eliminate atomic collisions in the initial structures. The whole process is divided into two parts, the steepest descent and the conjugate gradient method with 500 steps respectively. The initial structures of the systems were stable after energy minimization, and the reaction time of 50 ps was used to raise the temperature of the simulated reaction from 0 to 300 K. After heating, the simulated systems were then treated with 50 ps of reaction time for density equilibration. Finally, the simulated systems were equilibrated with a constant pressure operation under NPT ensemble, with a constant pressure balance of 500 ps at 300 K. Constant pressure equilibration was the last step of system equilibration. After all, the thermodynamic parameters were stabilized, and then 200 ns molecular dynamics simulations were carried out for the three simulated systems. And the experimental data collection interval was set at 1 fs for each system. The entire simulation used a time step of 2 fs, and a Langevin thermostat^50^ with a collision frequency of 1 ps. The storage interval is 2 ps/interval and the total record structure is 2000 frames. The data were kept for further study and analysis.

### Trajectory analysis

The CPPTRAJ module of Amber22 was used for the trajectory analysis, which included calculations for the RMSD, RMSF, R_g_, SASA, and hydrogen bond analysis^51^. K-means clustering was also performed utilizing CPPTRAJ, ten representative structures were obtained from each system. The tunnel analysis were obtained using CAVER 3.0^52^.

### MM-PBSA calculation

The MM-PBSA method as applied to small molecule binding is an end-point method estimating the binding free-energy difference between the protein–ligand complex^53–56^. The single-trajectory approach is favored for its straightforward implementation and cancellation of covalent energy errors as conformations for the complex and separated receptor and ligand are based on shared configurations from MD simulations.

MM-PBSA is often used in tandem with the closely related Molecular Mechanics Generalized Born Surface Area (MM-GBSA) approach as both utilize the same set of inputs for the prediction of binding free energies with continum solvation^57–59^:

1 $$ \Delta {\text{G}}_{{{\text{bind}}}} = {\text{G}}_{{{\text{complex}}}} - {\text{G}}_{{{\text{receptor}}}} - {\text{G}}_{{{\text{ligand}}}} $$

2 $$ \Delta {\text{G}}_{{{\text{bind}}}} = \Delta {\text{H}}{-}{\text{T}}\Delta {\text{S}} $$

3 $$ \Delta {\text{H}} = \Delta {\text{E}}_{{{\text{ele}}}} + \Delta {\text{E}}_{{{\text{vdW}}}} + \Delta {\text{G}}_{{{\text{PB}}}} + \Delta {\text{G}}_{{{\text{SA}}}} $$

4 $$ \Delta {\text{G}}_{{{\text{SA}}}} = \gamma \Delta {\text{SASA}} + \beta $$

In our calculation, for the ionic strength, a value of 0.1 M was used, and for the dielectric constants of the solvent and the solute, values of 80.0 and 1.0 were used, respectively^60,61^, 500 snapshots were extracted from the final trajectory for MM/PBSA calculation.

### Allosteric path analysis

**Figure 2:**
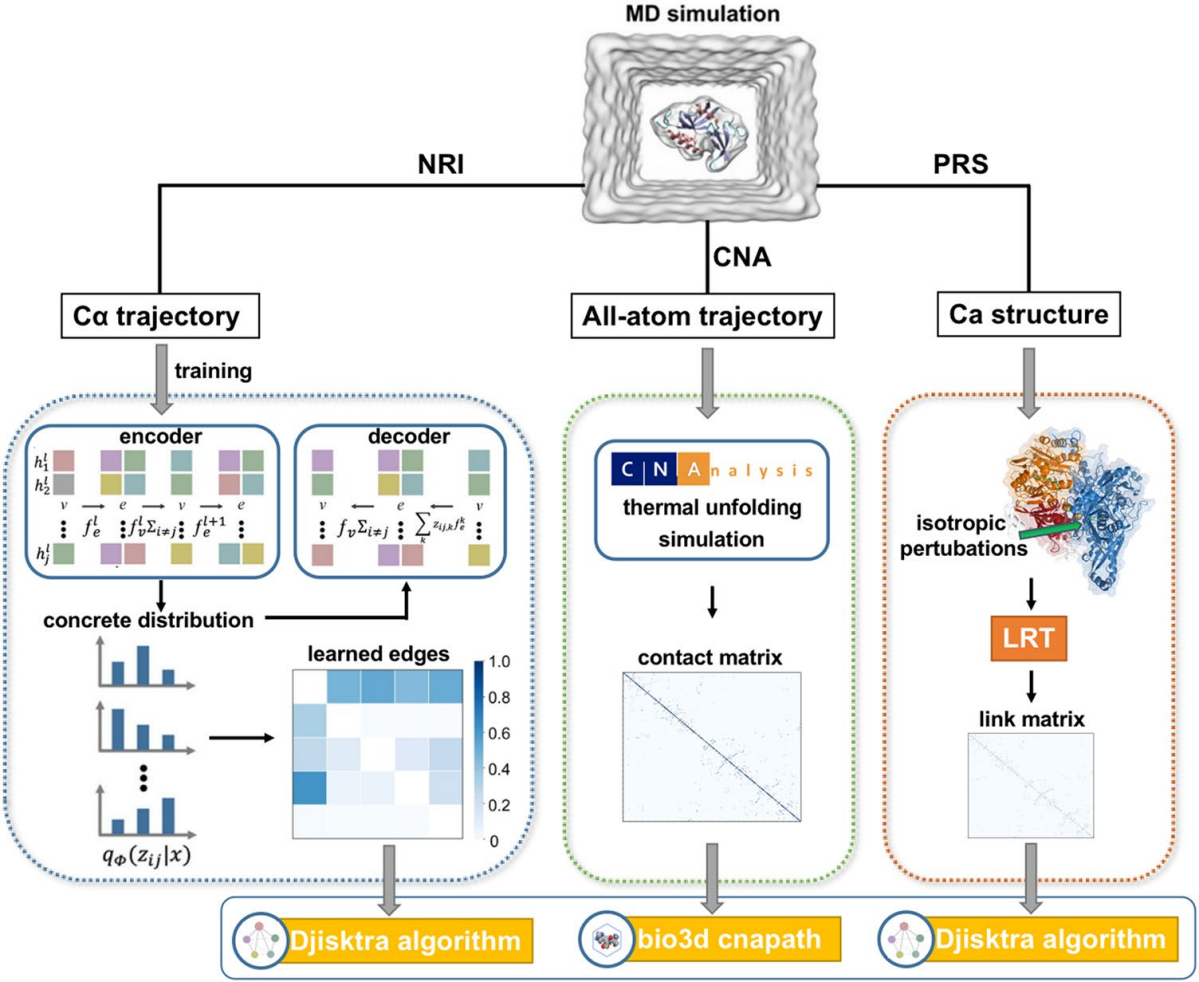
The work flow of allosteric path analysis.

The work flow of allosteric path analysis was shown in Fig. 2. NRI-MD^28^ (https://github.com/juexinwang/NRI-MD) was used to inferred residue-level and domain-level interaction, and then used Djisktra algorithm to list shortest allosteric path. NRI-MD inferred the degree of interaction between residues from the simulated trajectory of the protein through unsupervised learning, which is achieved by obtaining hidden variables in the process of trajectory reconstruction. These latent variables are the low-dimensional spatiotemporal characteristic abstracted from protein trajectories, which can well reflect the internal relationship between residues. By accumulating the residue interactions within the domain, the domain-domain interaction profile of the protein can be obtained. Since NRI-MD's current algorithm cannot support protein trajectories with more than 150 residues, we sampled XO's protein every 10 residues, and the original 1332 residue-sized protein was compressed to 134 residues. Next we divided 2000-frames of XO's trajectory into train, validate, and test sets at intervals of 30, 40, and 50 frames, respectively. The default encoder and decoder of the model are multilayer perceptron (MLP) and recurrent neural networks (RNN) were used, respectively.

We used first and half frame of all-atomic trajectory as an ensemble of networks for thermal unfolding simulation to produce a contact matrix by CNA, and then use Bio3D^62^cnapath to generate corresponding allosteric paths. CNA simulates the unfolding process of the protein by removing the covalent bonds in the protein, thereby obtaining the residue contacts in the protein.

Perturbed response Scan (PRS) excels in identifying residues imperative for conformational shifts and inter-residue communication. We implemented PRS based on Python rather than prody, we also added the ability to point out key residues and visualize them. In PRS, we use the Fibonacci algorithm to generate 100 unit forces uniformly dividing the three-dimensional space and apply them to the disturbance sites to ensure isotropic perturbations, and then use LRT to screen the associated residues, and finally use the Djiskra’s algorithm to generate the shortest paths.

## Results and discussion

### The binding mode of inhibitors to XO

The binding poses of ALPM and LWM were determined by molecular docking, shown in Fig. 1B and C. The binding force between ALPM and XO consists of Van der Waals forces provided by E652, T653, V654, V663, G664, I666, K771, F775, K807, L834, N869, S907, N908, T909, hydrogen bonds provided by T646, T803, R804, D872, alkyl and pi–alkyl forces by C662, H665, L873 and charge attraction provided by D872. While the binding force between LWM and XO consists of Van der Waals forces provided by L648, V654, I666, F775, T803, R804, H849, E868, N869, N908, T909, A910, hydrogen bonds provided by T646, E652, T653, K771, D872, S907, alkyl and pi–alkyl forces by H665, L807, L873 and charge attraction provided by E652.

### Structural stability and dynamics properties of the four systems

**Figure 3:**
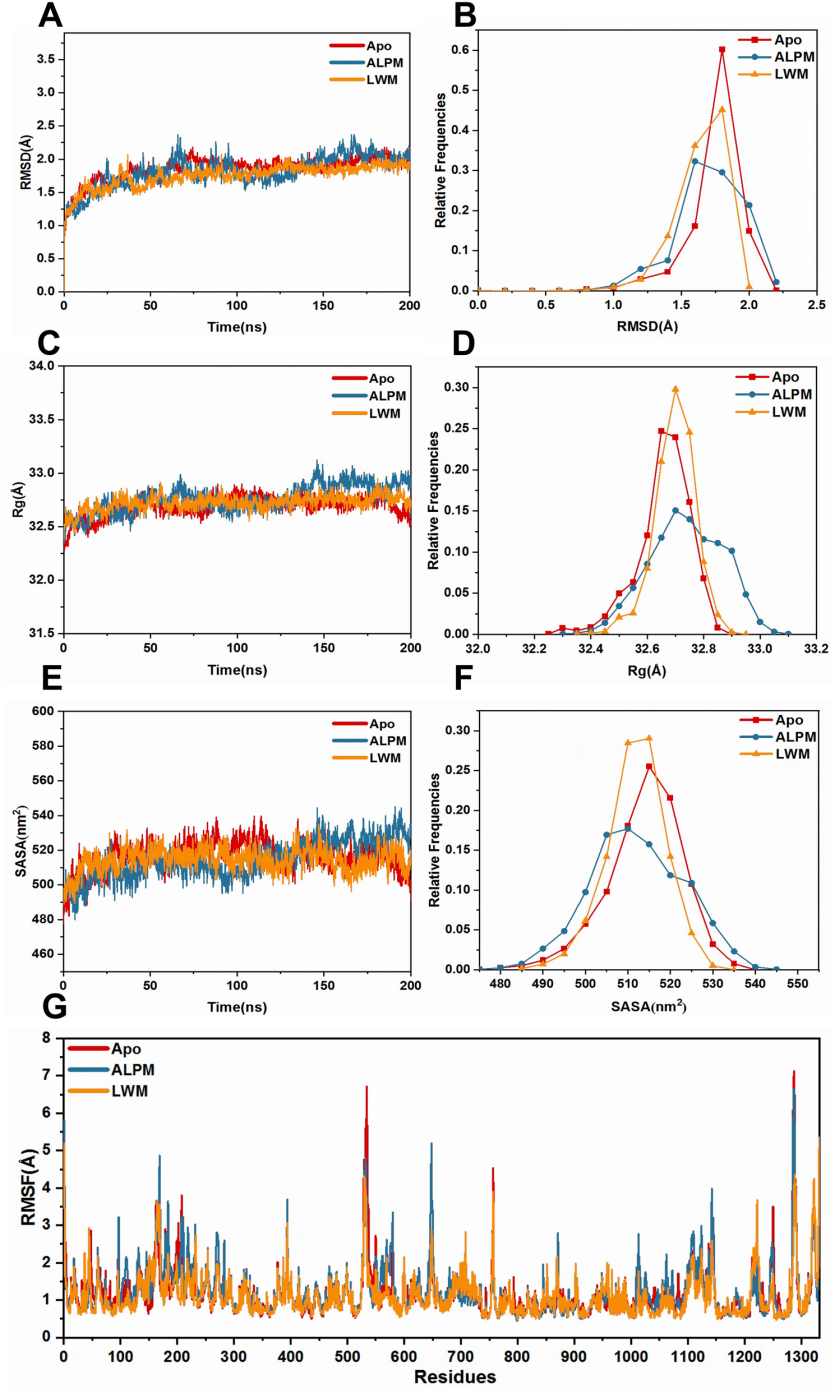
Analysis of structure stability. (**A**)The temporal evolution of the RMSDs from their initial structure of the three systems. (**B**) Relative Frequencies of RMSDs. (**C**) Radius of gyration over 200 ns MD for the three systems. (**D**) Relative Frequencies of radius gyration. (**E**) SASA over 200 ns MD. (**F**) Relative Frequencies of SASA. (**G**) The RMSFs of the CA atoms.

Following the generation of MD trajectories, the stability of simulations was assessed by calculating the root mean square deviation (RMSD) of CA atoms (as shown in Fig. 3A and B). After approximately 70 ns of simulations, equilibrium was reached in the RMSD of all three MD trajectories, suggesting the stability of all systems. However, in the the ALPM system, a broader attribution was noted, hinting at its lower stability in comparison to the other systems and more significant structural changes. Additionally, the minor mean RMSD values under 3 Å indicated no significant conformational changes in these systems. In summary, the equilibrated 200 ns trajectories were deemed suitable for further analysis.

The R_g_ (radius of gyration) value, a indicator of the overall size and compactness of the protein conformation, was found to be higher for the ALPM system post 150 ns compared to the other systems. This suggested a larger volume for the ALPM in comparison to the other complexes (as shown in Fig. 3C and D). Furthermore, the broader attribution in the ALPM system indicated its lower stability compared to the other systems.

The solvent-accessible surface area (SASA) is used to estimate the number of residues present in the surface regions of the protein and the number of residues that are in the hydrophobic core, which are buried. As shown in Fig. 3E and F, SASA values was also found to be higher for the ALPM system post 150 ns in comparison to the other systems. Further more, the distribution of SASA in ALPM systems was also broader than that in Apo and LWM systems, which consistent with that of the R_g_ values.

Then we calculated RMSF values of CA atoms for the three systems to investigate stability. The results of RMSF were shown in Fig. 3G. LWM exhibited a lower RMSF than both Apo and ALPM, indicating better rigidty of XO after binding with LWM peptide. Also, reducing protein flexibility may be one reason why LWM peptide has better inhibitory activity than ALPM peptide.

### Tunnel analysis

**Figure 4:**
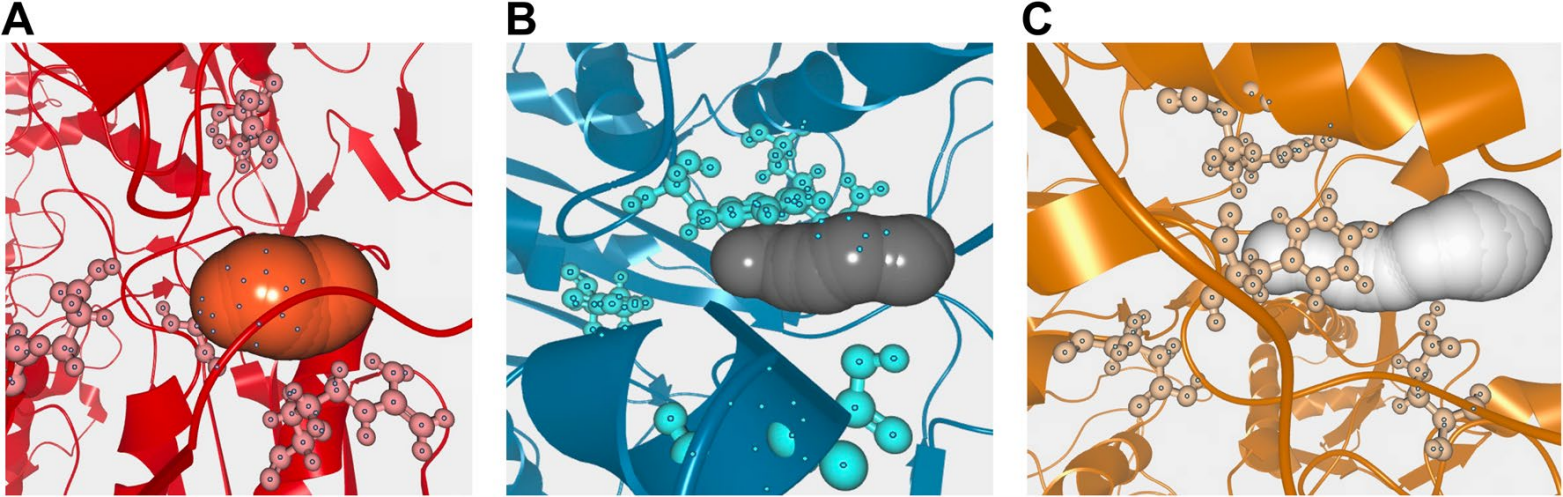
Representation of tunnels. (**A**) Apo. (**B**) ALPM. (**C**) LWM.

The tunnel is the access of the substrate to the catalytic site. Here, we used the F914, R880, E802 and E1261 residues to locate the active site for tunneling analysis of representative conformations of the three systems obtained by clustering. From the results shown in Fig. 4 and Table 1.

**Table 1:** The result of tunnels analysis for three systems.

System	Bottleneck radius(Å)	Tunnel length(Å)
Apo	2.10	3.85
ALPM	1.77	7.88
LWM	1.76	12.18

We can find that the tunnels became longer and narrower after combination of both inhibitors, with LWM of 12.18 Å, being longer and more tortuous than ALPM and Apo. Such a change is unfavorable for substrate to entry and may be the mechanism by which these two inhibitors are able to non-competitively inhibit XO.

### Analysis of the interaction between protein and inhibitors

The results of MM-PBSA were shown in Table 2. The binding free energy of ALPM was − 13.89 ± 1.02 kJ/mol, while the binding free energy of LWM was − 35.54 ± 0.95 kJ/mol, the binding free energy was lower, indicated a better affinity.

**Table 2:** The results of MM-PBSA.

System	ALPM	LWM
ΔE_vdW_	− 30.81 ± 1.49	− 40.52 ± 0.71
ΔE_ele_	− 198.46 ± 10.67	− 302.56 ± 4.49
ΔG_gas_	− 229.27 ± 11.95	− 338.11 ± 5.10
ΔG_solv_	215.38 ± 11.18	302.56 ± 4.49
ΔG_total_	− 13.89 ± 1.02	− 35.54 ± 0.95

**Figure 5:**
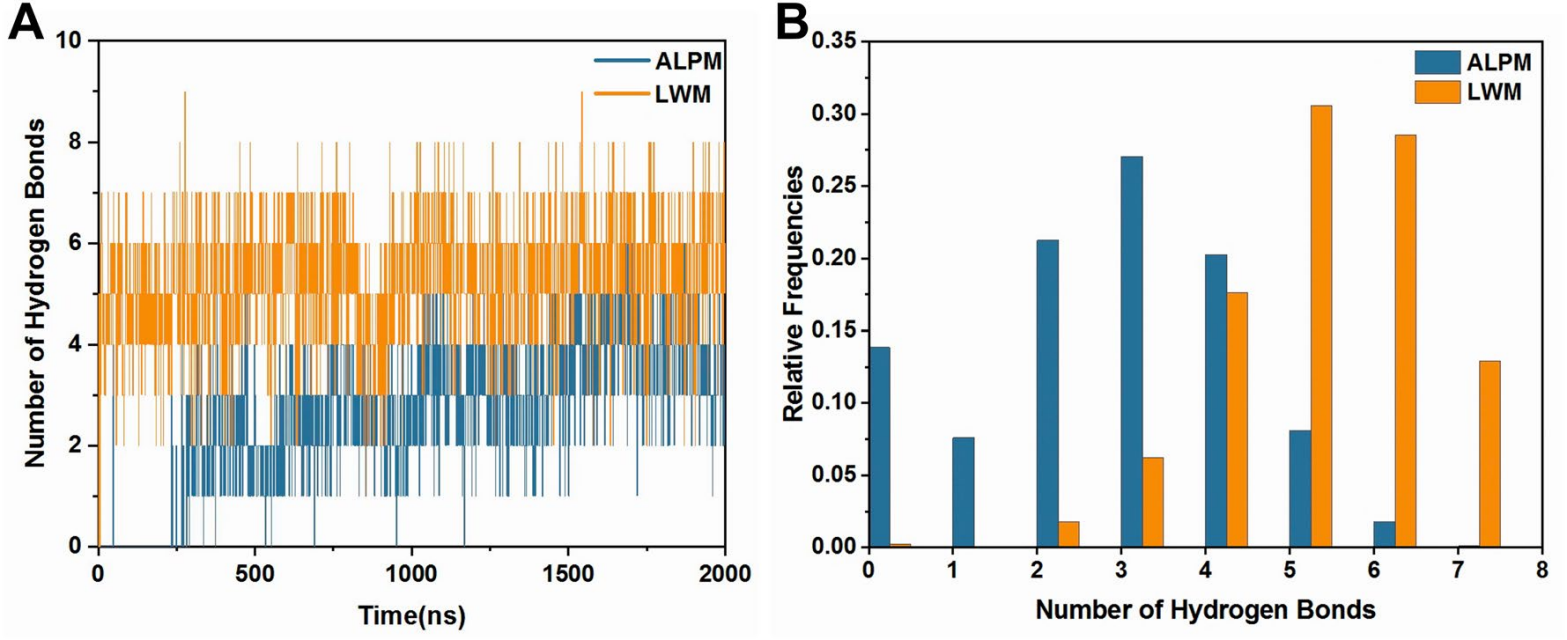
Analysis of hydrogen bonds. (**A**) Numbers of hydrogen bonds between receptor and inhibitors in the ALPM and LWM systems. (**B**) Frequency distribution of hydrogen bond numbers.

We also analyzed hydrogen bonds, shown in Fig. 5. The analysis showed that the more hydrogen bonds were formed between protein and inhibitor in LWM system during 200 ns simulation than ALPM system. This provided a stronger binding force for LWM peptide.

### Allosteric path analysis

**Figure 6:**
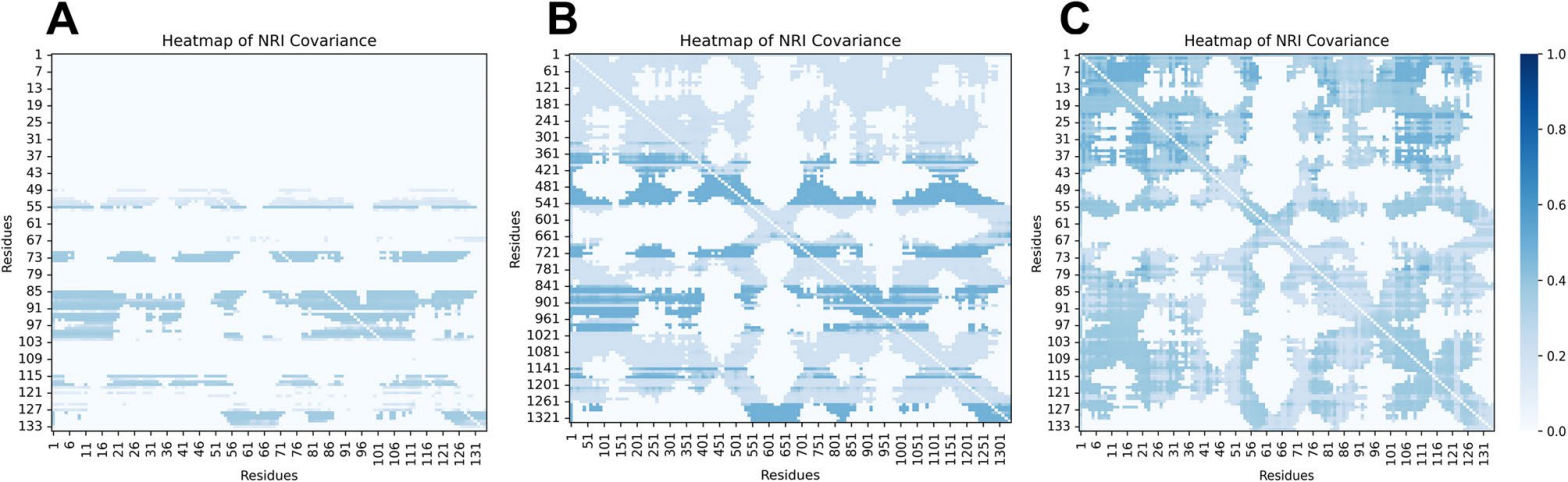
Heatmap of NRI covariance. (**A**) Apo. (**B**) ALPM. (**C**) LWM.

**Figure 7:**
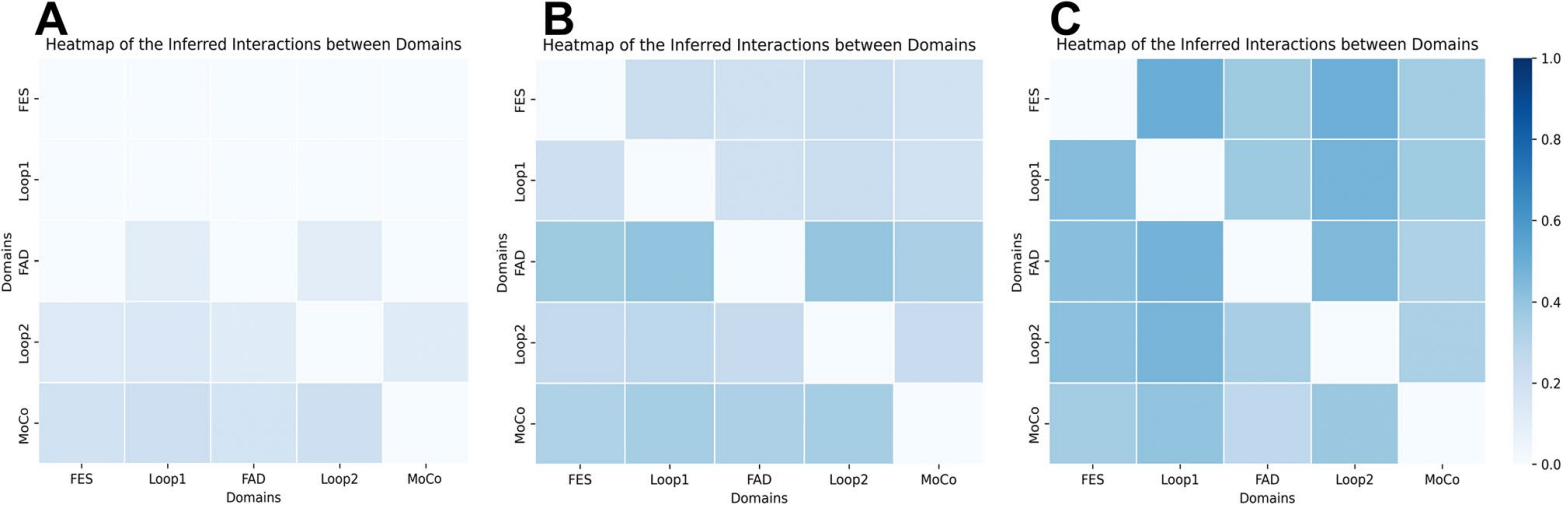
Heatmap of the inferred interactions between covariance. (**A**) Apo. (**B**) ALPM. (**C**) LWM.

To explore how binding events of the two non-competitive inhibitors affect the protein active pockets, and how they differ, we utilized multiple methods including deep learning. NRI-MD was used to inferred residue-level (Fig. 6) and domain-level (Fig. 7) interaction.

In the LWM system, the interaction between residues and that between domains was found to be the strongest, followed by the ALPM system, with the Apo system showing the least interaction. This suggested that the relatively looser conformation of the empty protein did not allow for significant internal interaction, whereas the internal interaction of the inhibited XO was enhanced with the increase in the inhibitory effect. In the empty protein state, domain interactions were predominantly concentrated between the loop2 and MoCo domains. The binding of the inhibitor, however, amplified interactions with the FES, Loop1, and FAD domains. This was consistent with the occurrence of allosteric binding or mutation in the NRI-MD article which increased interdomain communication. Furthermore, in the NRI-MD study, the accumulation of residue-to-residue interactions was described as an indicator-free energy fraction. The change in free energy fraction, before and after allosterism, was found to be linearly related to the experimentally measured free energy. Therefore, the combination of LWM and ALPM increased the free energy of the system, rendering the system relatively more unstable.

**Figure 8:**
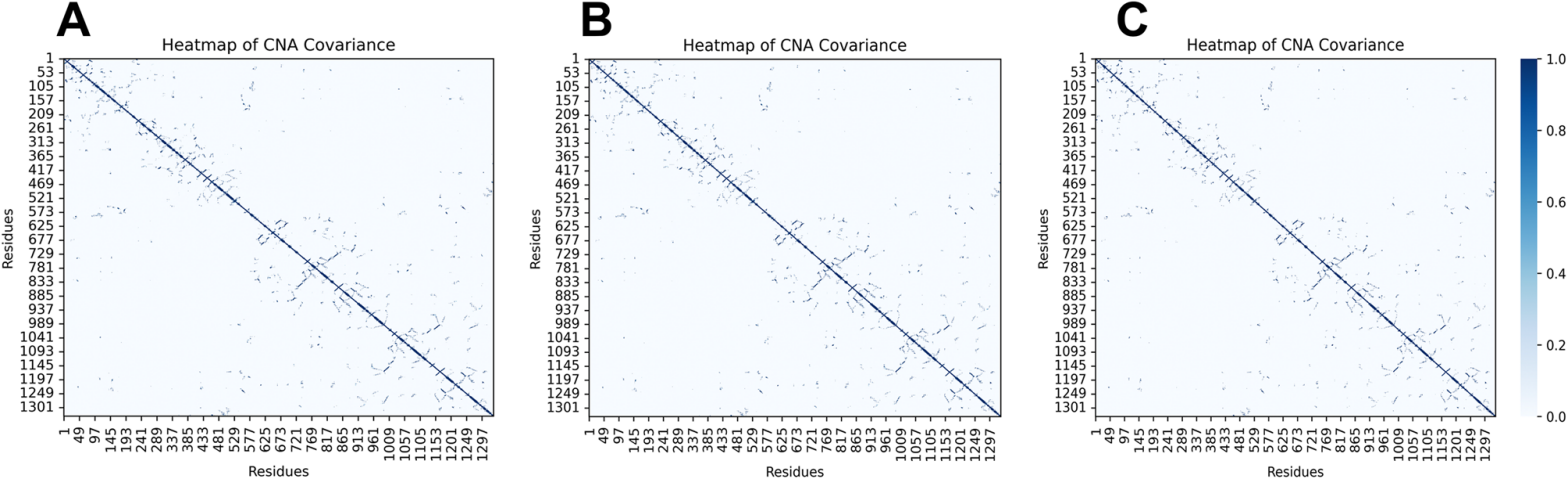
Heatmap of CNA covariance. (**A**) Apo. (**B**) ALPM. (**C**) LWM.

Then, constraint network analysis (CNA) was used to calculate neighbor stability maps, shown in Fig. 8. The stability maps reflect the local stabilities of the residue-residue contacts. Similar CNA maps revealed that the change of the active site was not simply the result of the changes of a few residues, but the accumulation of small energy and conformational changes of multiple residues.

**Figure 9:**
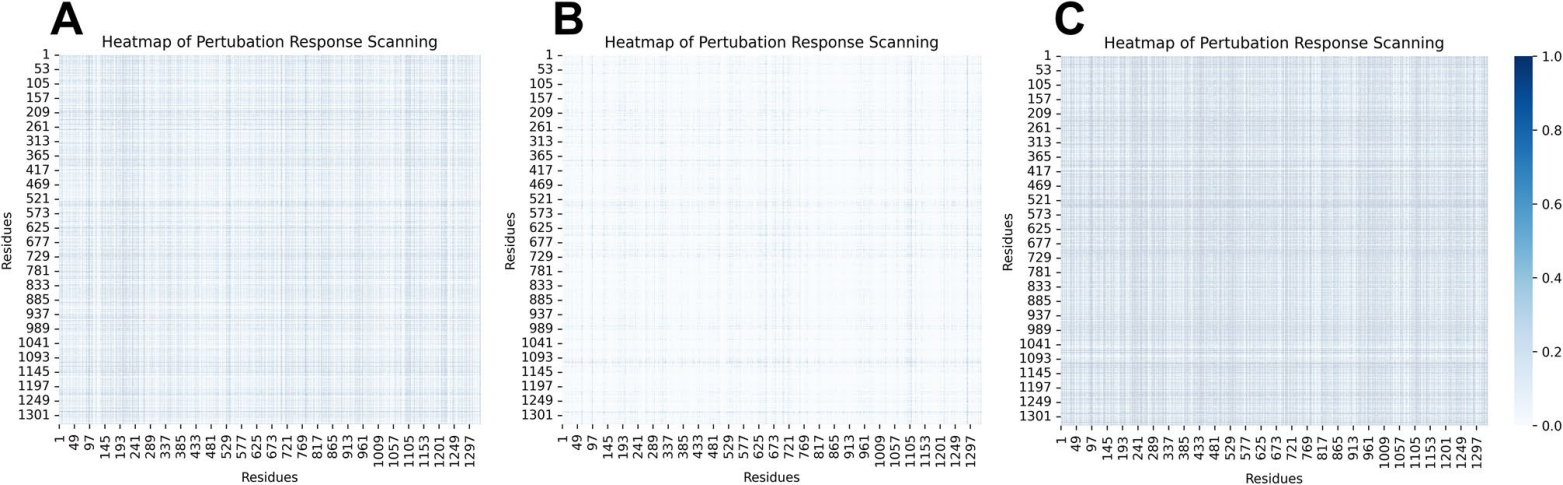
Heatmap of perturbation response scanning. (**A**) Apo. (**B**) ALPM. (**C**) LWM.

By applying isotropic perturbations to each residues, perturbation response scanning (PRS) were used to obtain the corresponding fluctuation response of the entire protein network. As can be seen in Fig. 9, the response to perturbation was stronger with LWM peptide inhibiting, while the response intensity decreased in ALPM and Apo. The results of the PRS were consistent with those of the NRI. This indicated that after the combination of the two allosteric inhibitors, the residues in the protein were more closely linked, which made the allosteric interaction affected the active center of the junction to achieve an inhibition effect.

**Figure 10:**
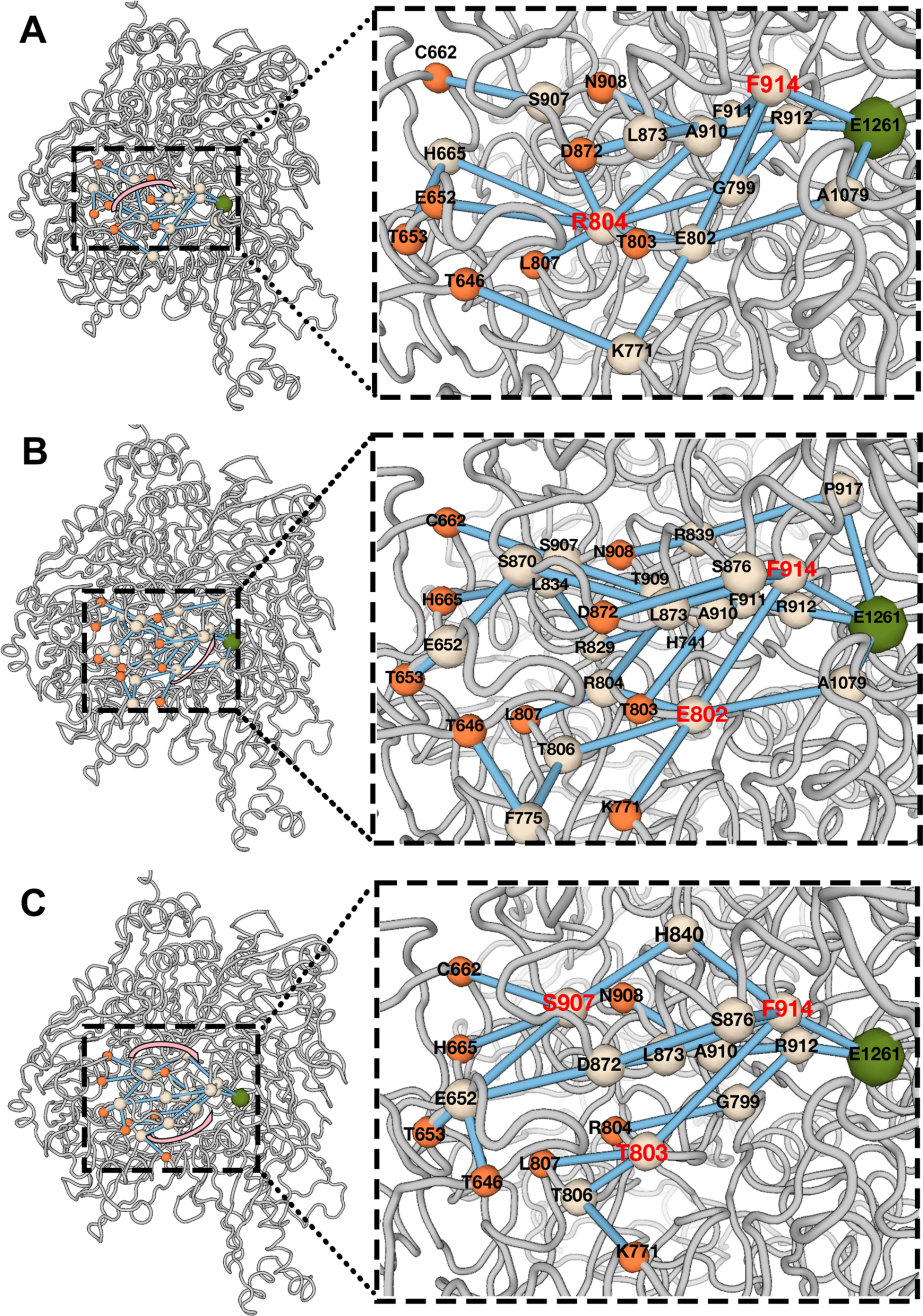
Conformational transition transduction pathways in proteins. The start nodes are orange, the middle nodes are white, and the end nodes are green. (**A**) Apo. (**B**) ALPM. (**C**) LWM.

Here, we generate allosteric paths using these three methods, and the path information is in the Table S1. Due to sampling was not precise enough, we did not plot the paths presented by NRI-MD and PRS, but picked the CNA results instead. The allosteric paths are shown in Fig. 10, the start nodes are orange, the middle nodes are white, and the end nodes are green. In Apo, the transfer path is relatively simple, and R804 and S914 are nodes through which multiple paths pass, which is more important. However, in ALPM, we can see relatively complicated and chaotic transmission paths, which may also lead to poor inhibition effect. Similarly, E802 and F914 show relatively important hub status. Finally, the path of LWM is clearly divided into two parts: the upper node is S907, and the lower node is T803, which converge to F914 and thus allosteric the active site.

F914 is an important conformational change transmission site since it was a most mediated site in the network of allosteric pathways from binding sites to active sites. Although F914 now has not been shown experimentally to be a key allosteric residue in other study about XO, but there were some works showed the prediction are consistent with the experiment. For example, Ohm (used perturbation propagation algorithm) predicted that R286 and E390 are the most critical residues in the Caspase-1 allosteric network, S332, S333, and S339 were the next most important residues that are in perfect accord with experimental results (mutated nine residues of Caspase-1 to test their roles in allosteric pathways)^63^. And PRS predicted the allosteric pathways in PIN1^64^. These successful examples proved that our reasoning has some reliability.

## Conclusions

In this study, we conducted 200 ns molecular dynamics simulations of Apo, LWM and ALPM systems and carried out a variety of analyses. The results show that LWM peptide and XO have stronger binding ability, which may be related to more hydrogen bonds. After binding to the inhibitor, the XO tunnel deforms, while LWM is thinner, longer and more tortuous than ALPM. In addition, studies of allosteric pathways have found that F914 may be a key residue in the allosteric path. Our study will provide new insights into food-derived anti-XO inhibitors and may contribute to the development of food drugs that reduce uric acid without side effects.

### Supplementary Information

Supplementary Table S1.

## Data Availability

PRS is implemented based on Python, and the detailed code can be available in https://github.com/heyigacu/PRS.
