# Supplementary material for: Using deep learning and molecular dynamics simulations to unravel the regulation mechanism of peptides as noncompetitive inhibitor of xanthine oxidase
Source: Sci Rep. 2024 Jan 2;14:174. doi: 10.1038/s41598-023-50686-0 (PMC10761953; doi:10.1038/s41598-023-50686-0)
Supplement: Supplementary file 1 — Supplementary Table S1. [file 41598_2023_50686_MOESM1_ESM.docx]

Using Deep Learning and Molecular Dynamics Simulations to Unravel the Regulation Mechanism of Peptides as Noncompetitive Inhibitor of Xanthine Oxidase

Yi He^1,†^, Kaifeng Liu^1,†^, Fuyan Cao^1,†^, Renxiu Song^1^, Jianxuan Liu^2^, Yinghua Zhang^2,^*, Wannan Li^3,^* and Weiwei Han^1,^*

^1^Key Laboratory for Molecular Enzymology and Engineering of Ministry of Education, School of Life Sciences, Jilin University, Changchun 130012, Qianjin road 2699, China.

^2^Jilin Academy of Chinese Medicine Sciences，Changchun 130012, Chuangju road 155, China.

^3^Edmond H. Fischer Signal Transduction Laboratory, School of Life Sciences, Jilin University,
Changchun 130012, Qianjin road 2699, China.

***** Correspondence: [zhangyinghua0214@126.com (Y.Z.),](mailto:zhangyinghua0214@126.com,) [liwannan@jlu.edu.cn](mailto:liwannan@jlu.edu.cn) (W.L.), [weiweihan@jlu.edu.cn (W.H.),](mailto:weiweihan@jlu.edu.cn,) Phone: +86(431)85155218.

^†^ These authors contributed equally to this work.

Table S1: Allosteric path by different methods. Toplogies and MD trajectories are available on the dropbox (https://www.dropbox.com/scl/fo/wzsb5qan0kkj1crmh1c5m/h?rlkey=7jsiclqsf2bt60km25ti3q1a6&dl=0).

| **System** | **Apo** | **Apo** | **Apo** |
| --- | --- | --- | --- |
| **Method** | **NRI** | **CNA** | **PRS** |
| THR646 | 641->1331->1261 | 646->771->802->1079->1261 | 641->644->652->868->840->914->1261 |
| GLU652 | 651->1261 | 652->804->802->914->1261 | 651->869->866->878->914->1261 |
| THR653 | 651->1261 | 653->665->804->799->912->1261 | 653->647->870->874->914->1261 |
| CYS662 | 661->1261 | 662->907->804->910->912->1261 | 662->868->840->914->1261 |
| HIE665 | 661->1261 | 665->804->799->914->1261 | 665->835->800->910->798->1261 |
| LYS771 | 771->1261 | 771->802->1079->1261 | 771->802->798->1261 |
| THR803 | 801->1261 | 803->802->1079->1261 | 803->800->911->798->1261 |
| ARG804 | 801->1261 | 804->910->912->1261 | 804->801->798->1261 |
| LEU807 | 811->1261 | 807->804->799->912->1261 | 807->747->795->798->1261 |
| ASP872 | 871->1221->1261 | 872->873->911->914->1261 | 872->876->914->1261 |
| LEU873 | 871->1221->1261 | 873->911->914->1261 | 873->877->916->1261 |
| SER907 | 911->1221->1261 | 907->804->802->1079->1261 | 907->804->910->798->1261 |
| ASN908 | 911->1221->1261 | 908->910->912->1261 | 908->800->912->1261 |
| **System** | **LWM** | **LWM** | **LWM** |
| THR646 | 641->651->1261 | 646->652->907->840->914->1261 | 646->872->877->913->1261 |
| GLU652 | 651->1261 | 652->872->873->914->1261 | 652->871->874->915->911->1261 |
| THR653 | 651->1261 | 653->652->872->876->914->1261 | 653->647->871->874->910->916->1261 |
| CYS662 | 661->1261 | 662->907->840->914->1261 | 662->906->838->911->1261 |
| HIE665 | 661->1261 | 665->907->840->914->1261 | 665->807->801->798->913->1261 |
| LYS771 | 771->1261 | 771->806->803->914->1261 | 771->766->794->1039->1197->1264->1261 |
| THR803 | 801->1261 | 803->914->1261 | 803->800->740->911->1261 |
| ARG804 | 801->1262 | 804->799->912->1261 | 804->910->916->1261 |
| LEU807 | 801->1263 | 807->803->914->1261 | 807->665->837->739->911->1261 |
| ASP872 | 871->841->1261 | 872->876->914->1261 | 872->867->878->916->1261 |
| LEU873 | 871->841->1261 | 873->914->1261 | 873->914->911->1261 |
| SER907 | 911->841->1261 | 907->840->914->1261 | 907->840->739->911->1261 |
| ASN908 | 911->841->1261 | 908->910->912->1261 | 908->738->1205->1265->1261 |
| **System** | **ALPM** | **ALPM** | **ALPM** |
| THR646 | 641->571->1261 | 646->775->806->802->1079->1261 | 646->652->869->873->914->1261 |
| GLU652 | 651->1261 | 652->870->873->914->1261 | 652->870->873->878->916->1261 |
| THR653 | 651->1261 | 653->652->870->873->914->1261 | 653->645->654->807->747->797->912->1261 |
| CYS662 | 661->1261 | 662->907->909->911->1261 | 662->869->873->909->912->1261 |
| HIE665 | 661->1261 | 665->834->829->741->912->1261 | 665->836->839->913->1261 |
| LYS771 | 771->1261 | 771->802->914->1261 | 771->767->1077->1080->1261 |
| THR803 | 801->1261 | 803->910->911->1261 | 803->909->912->1261 |
| ARG804 | 801->1261 | 804->802->1079->1261 | 804->800->912->1261 |
| LEU807 | 801->1261 | 807->804->873->914->1261 | 807->803->767->798->1261 |
| ASP872 | 871->1261 | 872->876->914->1261 | 872->877->916->1003->1261 |
| LEU873 | 871->1261 | 873->914->1261 | 873->909->912->1261 |
| SER907 | 911->1081->1261 | 907->909->911->1261 | 907->839->911->1261 |
| ASN908 | 911->1081->1261 | 908->839->917->1261 | 908->800->912->1261 |
